# Yellow Fever in the South

**Published:** 1853-10

**Authors:** 


### YELLOW FEVER IN THE SOUTH.

It is gratifying to learn that the pestilence is abating in New Orleans; but while it is subsiding there it is spreading into the towns and villages around. The *New Orleans Bulletin* of the 13th September, says:

"The weather continues to be unfavorable in the highest degree. We doubt if such weather has ever prevailed before in any portion of the earth—so unhealthy, uncongenial, and unnatural—for three months at a stretch. As we write, a heavy, cold rain, is falling, and the sky wears a dark and sombre hue, that might suit December in its worst moods, but which is wholly unsuited to September, in this latitude. Notwithstanding the bad weather the mortality continues to decrease. We want no better evidence of the general subsidence of the epidemic, and no surer proof that the city will be, very shortly, entirely exempt from yellow fever, if strangers and absentees keep away for a few weeks longer. Let them stay away until they can return in safety. If they come back now they will be tempting food for the fever, and will, in all probability, fall victims."

The *Vicksburg Whig*, in reference to the epidemic, says:

"We feel that a high public and social duty makes it necessary for us to urge in the most earnest manner upon those of our citizeus absent from the city not to return, or visit it, unless compelled to do so, until they have full information as to the abatement of the epidemic. The fever has been, within the last week, not only an epidemic, but is becoming, if it has not already become so, a pestilence of the most fearful character. It is now more general and fatal than it was in 1841. It selects its victims without distinction. The old and the young, the rich and the poor, man in the fullness of his strength, and woman in the pride of youth, beauty, and loveliness, are alike its victims. It spares no ages, sexes, or conditions; not even the negroes, who have heretofore escaped. There is scarcely a tenanted house in the city not afflicted by the visitation to some extent. The mortality for the last few days has been fearful for the size of our present population, and the number of patients under treatment and convalescent, much larger than ever before known."

This was written on the 13th of September. Two days later the same paper has the following:

"We regret that we are not able to chronicle any abatement or decline of the pestilence. On the contrary, it will be seen from the Sexton's report for the last two days, that it is marching onward with fearful terror, and seems to gather strength and power as it progresses in its work of distress and desolation. As it grasps its victims it acquires a fresh love for food, and seizes each new one within its influence with as savage fierceness as the last. Other neighboring cities and towns (and even the country, in the county of Claiborne) are similarly afflicted. In Port Gibson, Grand Gulf, Yazoo City, etc., we learn that it rages with like violence, and that the citizens are fleeing in every direction. Even in Jackson and Raymond we are told that it has made its appearance, and created the greatest consternation."

